# Biomarkers in Small Intestine NETs and Carcinoid Heart Disease: A Comprehensive Review

**DOI:** 10.3390/biology10100950

**Published:** 2021-09-23

**Authors:** Markos Kalligeros, Leonidas Diamantopoulos, Christos Toumpanakis

**Affiliations:** 1 Department of Medicine, Warren Alpert Medical School of Brown University, Providence, RI 02903, USA; markos_kalligeros@brown.edu; 2 Department of Medicine, University of Pittsburgh Medical Center, Pittsburgh, PA 15213, USA; diamantopoulosln@gmail.com; 3 Neuroendocrine Tumor Unit, Centre for Gastroenterology, ENETS Centre of Excellence, Royal Free Hospital NHS Foundation Trust, London NW3 2QG, UK

**Keywords:** NET, neuroendocrine, biomarkers, small intestine NETs, carcinoid heart disease, NETest

## Abstract

**Simple Summary:**

Neuroendocrine tumors (NET), a heterogeneous group of tumors arising from neuroendocrine cells, often pose a diagnostic and therapeutic challenge for the clinician. Biomarkers can serve as a useful diagnostic, prognostic, and predictive tool in the management of these rare tumors. For years the field of NET biomarkers was mainly based on products se-creted by neuroendocrine tumor cells, however, during the last decade the development of nov-el multianalyte biomarkers has rapidly evolved the field. The aim of this review is to summa-rize the literature on the use and limitations of available NET biomarkers for the diagnosis and management of small intestine neuroendocrine tumors (SI-NETs) and carcinoid heart disease.

**Abstract:**

Biomarkers remain a valuable tool for the diagnosis and management of Neuroendocrine tumors (NETs). Traditional monoanalyte biomarkers such as Chromogranin A (CgA) and 5-Hydrocyondoleacetic acid (5-HIAA) have been widely used for many years as diagnostic, predictive and prognostic biomarkers in the field of NETs. However, the clinical utility of these molecules often has limitations, mainly inherent to the heterogeneity of NETs and the fact that these tumors can often be non-secretory. The development of new molecular multianalyte biomarkers, especially the mRNA transcript based “NETest”, has rapidly evolve the field and gives the ability for a “liquid biopsy” which can reliably assess disease status in real time. In this review we discuss the use of established and novel biomarkers in the diagnosis and management of small intestine NETs and carcinoid heart disease.

## 1. Introduction

Neuroendocrine neoplasms (NENs) first described as “karzinoide” by Dr. Oberndorfer in 1907 [1], comprise a family of heterogeneous tumors which can range from well differentiated neuroendocrine tumors (NETs) to poorly differentiated neuroendocrine carcinomas (NECs) [2]. NENs emerge from the diffuse endocrine system [3] and therefore can be found throughout the body, but they most commonly occur in the gastrointestinal tract with a peak incidence around the 5th and 6th decade of life [4,5]. Based on the latest Survival Epidemiology and End Results data, incidence of NENs is on the rise with almost 7 cases per 100,000 persons in the United States [6]. NENs can be alternatively classified as functional or non-functional based on the release of specific hormones by the tumor cells (e.g., serotonin, vasoactive intestinal peptide, insulin, gastrin, somatostatin, glucagon) and the subsequent development of various secretory syndromes.

Small intestine NETs (SI-NETs) (i.e., jejunal and ileal) comprise 42% of the tumors of the small intestine, while their incidence in the United States is 1.05 per 100,000 persons [7]. SI-NETs can be functional or nonfunctional, while their diagnosis is challenging and often occurs at an advanced disease stage as SI-NETs often remain asymptomatic or present with chronic abdominal pain. In rare cases initial presentation can be a complication which can include obstruction, perforation, or bleeding [8]. When SI-NETs are suspected, the combination of imaging and biomarkers can point towards diagnosis [9]. Multi-phase CT scan of the abdomen can serve as an initial diagnostic modality, while MRI can detect liver metastases with higher accuracy compared to CT. However, given that the majority of these tumors have somatostatin receptors, functional imaging modalities such as PET/CT with gallium-68–labeled somatostatin analogs have dramatically improved the diagnostic sensitivity [10]. In addition, biomarkers such as Chromogranin A (CgA) and 24-h urinary 5-Hydrocyondoleacetic acid (5-HIAA) have been widely used as SI-NETs diagnostic aids, however with several limitations and suboptimal diagnostic accuracy [11]. Despite the undeniable importance of imaging and biomarkers, SI-NET diagnosis can only be established with tissue biopsy and further tumor grading based on the combination of Ki-67 proliferative index with cell mitotic rate [11].

Carcinoid syndrome can develop in up to 40% of patients with NETs and is a result of vasoactive substances overproduction, mainly serotonin, by tumor cells [12]. Clinical manifestations range from the classic triad of cutaneous flushing, diarrhea and bronchospasm to neuropsychiatric symptoms, pellagra and local or distant fibrosis [13]. The liver normally exerts a first pass metabolism on tumor-secreted vasoactive substances and prevents them from entering systemic circulation. Thus, carcinoid syndrome is not developed until tumor burden is profound or, most commonly, liver metastases occur. Carcinoid heart disease (CHD) is a fibrotic complication of carcinoid syndrome and can affect up to 40% of these patients [14]. CHD is a detrimental complication of elevated circulating levels of vasoactive substances such as serotonin and its metabolites, which produces fibrosis and endocardial plaque formation mainly on right sided cardiac valves and endocardium, subsequently leading to right sided heart failure [15]. Interestingly, in most cases, left sided heart failure is avoided because 5HIAA is inactivated in the lungs [13]. However, in patients with patent foramen ovale (PFO) with right-to-left shunt, bronchial NETs or very high serotonin levels, left-sided CHD can develop as well [14]. Unfortunately, the presence of CHD is associated with substantial morbidity and mortality.

In this review we delineate the use of biomarkers in the diagnosis, prognosis and management of NENs with focus on neuroendocrine tumors of the small intestine (SI-NETs). Additionally, we aim to explore the use of biomarkers in assessing the diagnosis and management of carcinoid heart disease.

## 2. Materials and Methods

PubMed was searched using the terms “neuroendocrine tumor”, “neuroendocrine neoplasm”, “midgut”, “small intestine”, “small bowel”, “biomarkers”, “chromogranin A”, “5-HIAA”, “NETest”, “carcinoid syndrome”, carcinoid heart disease” to identify articles in English referring to SI-NETs, carcinoid syndrome, carcinoid heart disease and their respective biomarkers. No systematic search/review of the literature was performed. We extracted information including the name of the biomarker, category of biomarker (single-analyte vs. multi-analyte), as well as type of biomarker based on its correlation with natural history of disease and symptoms (type 0), effects of an intervention (type 1), clinical benefit and disease prognosis (type 2) as per National Institutes of Health classification [16], separately for SI-NETs and CHD respectively. Article references considered to be relevant to the topic were also selected and reviewed and their data were extracted in the above fashion. No statistical analysis of data was performed, and we focused on most recent literature results and meta-analyses where feasible.

## 3. Biomarkers in the Diagnosis and Management of Small Intestine NETs (SI-NETs)

**Figure 1:**
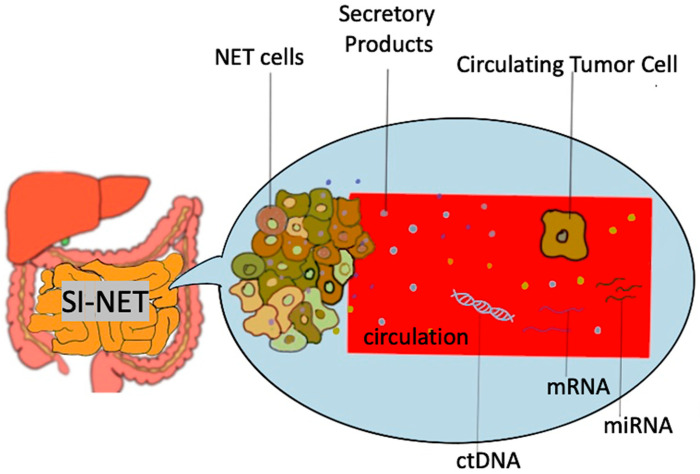
Schematic representation of biomarkers used in SI-NET. Secretory products include biomarkers such as Chromogranin A, 5-hydroxy-indole acetic acid, pancreastatin etc.; mRNA, messenger RNA, miRNA, microRNA, SI-NET, small intestine neuroendocrine tumor; ctDNA, circulating tumor DNA.

Biomarkers used in the setting of SI-NETS are extrapolated from the general field of GEP-NETs and other NETs and are divided in two main categories: mono-analyte and multi-analyte biomarkers [17] (Figure 1). The former are specific molecules (protein, gene, RNA) whose levels in the blood or other bodily fluids can be used to predict the presence of disease as well as assess its degree of severity. This is true for traditional biomarkers like chromogranin A and 5-HIAA, as well as biomarkers used in other malignancies (e.g., CEA for adenocarcinoma). The main disadvantages of the single-analyte method include the high heterogeneity of NETs, the lack of secretory products in high proportion of NETs as well as the lack of standardization in measurement of biomarker concentrations, which subsequently lead to a range of sensitivities and specificities across different assays used [18]. To address the inherent limitations of mono-analyte assays, there has been a gradual shift towards the development of tests that are able to identify multiple genomic regulators (mRNA, miRNA, ctDNA) associated with a specific disease process, with NETest being the most heavily investigated multi-analyte assay in the field of NETs [19]. A brief overview of SI-NET biomarkers is presented on Table 1.

### 3.1. Mono-Analytes

#### 3.1.1. Chromogranin A (CgA)

CgA is still one of the most implemented biomarkers in the diagnosis and prognosis of NETs. This biomarker is an acidic, hydrophilic glycoprotein found in large dense core vesicles of neuroendocrine cells. As such, elevated serum CgA levels in the appropriate clinical context may serve as a useful diagnostic marker of neuroendocrine neoplasms [20]. A recent metanalysis found that the diagnostic sensitivity of CgA in NETs is 73% and its specificity 95% [21]. However, serum CgA accuracy varies based on the type of NET and it is considered somewhere between 40–70% in patients with SI-NETs [11]. In addition to that, predictive value of serum CgA in terms of disease progression and response to treatment seems to be limited in SI-NETs, while certain studies have demonstrated that CgA may not be a robust marker of disease recurrence [22]. From a prognostic standpoint elevated CgA (>6× the upper normal limit) has been associated with worse outcomes in patients with SI-NETs [23], while the concentration of CgA correlates with the tumor differentiation and burden [24]. Pitfalls of CGA include false elevation in the presence of various conditions including atrophic gastritis, renal disease, inflammatory bowel disease (IBD), liver disease as well as following treatment with proton pump inhibitors [25,26]. Moreover, significant variations of CgA levels between different assays have been reported [27] and measurements in the same lab or with the same assay are recommended. Based on the above limitations, the clinical utility of CgA as a biomarker for SI-NETs is gradually declining [28].

#### 3.1.2. 5-Hydroxyindoleacetic Acid (5-HIAA)

5-HIAA is a breakdown product of serotonin and can be measured either in a urinary or serum sample. 5-HIAA has shown great sensitivity for jejuno-ileal tumors and carcinoid syndrome. The downside of the urine 5-HIAA includes the need for a 24-h urine collection which is time consuming and also prone to sampling errors. In this regard, serum measurement of 5-HIAA from a single lab draw has started replacing the traditional urinary sampling. Plasma 5-HIAA (sensitivity 89–95%; specificity 75–85%) is an alternative to the 24 h urine 5-HIAA (sensitivity 35–70%; specificity up to 100%), which can be affected by multiple foods and drugs. False-negative results are also possible in patients with renal insufficiency and inadequate urine production [29]. Additionally, 5-HIAA becomes positive in late disease stages and it does not correlate well with disease progression [29]. In terms of prognostic value in SI-NETs, Laskaratos et al. found that baseline urinary 5-HIAA greater than 10× the upper limit was associated with worse overall survival (OS), while persistently low urinary 5-HIAA with increased OS [30]. Similarly in a study by Schrivers et al., low urinary excretion of 5-HIAA was associated with improved OS in patients with disseminated midgut carcinoid tumors [31]. However, in a study by Zandee et al. 5-HIAA greater than 10× the upper limit was associated with worse OS only in a univariate model but lost its prognostic significance in the multivariate model [32]. Thus, the role of 5-HIAA as a prognostic marker in SI-NETs is not clear.

#### 3.1.3. Chromogranin B (CgB)

CgB shares similar characteristics with CgA but it is not affected by renal function and treatment with PPIs. Hence, it has been proposed as an adjunct CgA measurement in patients with decreased renal function or PPI use [33]. However, recent studies have shown that measurement of CgB in addition to CgA, does not add important information on the management of GEP-NETs [34].

#### 3.1.4. Pancreastatin

Pancreastatin is a breakdown product of CgA. Compared to CgA, and similar to CgB, pancreastatin can be more specific among patients receiving PPI, while increased levels have been associated with SI-NETs liver metastases [35]. A recent retrospective study by Tran et al., comprising of 218 SI-NET patients, showed that pancreastatin may also be superior to CgA in detecting SI-NET disease progression with an overall accuracy of 78.9% [36]. In addition, Woltering et al. studied pre and post-operative levels of pancreastatin in 300 patients with SI-NETs who underwent surgical cytoreduction. Authors found that patient who had normal pancreastatin after surgery had significantly better 5- and 10-year survival rates compared to patients in whom pancreastatin remained elevated postoperatively (*p* < 0.0001) [37]. Similar findings were also reported by Sherman et al. where higher pancreastatin levels were associated with worse PFS and OS in patients with SI-NETs [38].

#### 3.1.5. Neuron Specific Enolase (NSE)

NSE is a glycolytic enzyme that can be found in neurons and neuroendocrine cell cytoplasm [39]. Although it may be increased in poorly differentiated NET, it has no role in the diagnosis of SI-NETs which are usually moderate to well differentiated tumors [40]. From a prognostic standpoint NSE has been reported as an independent predictor of overall survival in patients with GEP-NETs, with higher levels correlating with worse OS [41].

#### 3.1.6. Neurokinin A (NKA)

Neurokinin A (NKA) belongs to the family of tachykinins and it is considered an accurate biomarker in terms of SI-NET prognosis. More specifically NKA has been used to monitor response to therapy with somatostatin analogs (SSAs) with increased levels of NKA after SSA treatment associated with worse overall survival [42].

#### 3.1.7. Paraneoplastic Antigen Ma2 Autoantibodies (PNMA2)

PNMA2 have been evaluated in the past as potential biomarkers for the diagnosis and recurrence of SI-NETs. Although available data are very limited, Cui et al. found that PNMA2 differentiated SI-NET patients from healthy controls with an AUC of 0.734 to 0.816, while PNMA2 was also superior to CgA in terms of predicting progression and disease recurrence [43]. However, no other studies have externally validated those findings.

### 3.2. Multi-Analytes

#### 3.2.1. Blood mRNA-Based NET Biomarker: “NETest”

NETest is a novel multianalyte biomarker developed by Wren Laboratories (Branford, CT, USA). Traditional mono-analyte biomarkers have variable sensitivity and specificity in diagnosing different NETs mainly due to the high heterogeneity of these tumors. Development of NETest was based on the identification of individual genes from tumor cells and whole blood samples from patients with NETs. The finalized NETest is now a standardized and reproducible biomarker that analyses 51 different NET transcripts [44]. NETest is performed by PCR and interpreted as a score between 0–100. A score of ≥20 correlates with NET diagnosis, while in patients with a known NET a NETest score >40 correlates well with disease progression [45]. In addition, it seems that factors such as patients’ demographics and concomitant use of specific medications (e.g., PPIs) do not impair the performance of the assay.

NETest has been associated with accurate diagnosis of GEP-NETs, including SI-NETs, as well as response to treatment and disease recurrence. The diagnostic accuracy of NETest for GEP-NET is considered to be around 95% [11]. Of note, NETest has shown promising results even in challenging clinical NET complications, such as Mesenteric Fibrosis, for which only radiological criteria have been used in the past. In their study Laskaratos et al. showed that NETest can detect fibrosis and aid in the diagnosis of this SI-NET complication with an accuracy of 100% [46]. Besides its diagnostic accuracy, there are promising signals that NETest can accurately differentiate between stable and progressive disease, as wells as response to treatment [47]. A positive NETest score after operative tumor resection is associated with disease recurrence [48], while a recent multicenter retrospective study with 153 patients, 62 of which had a SI-NET, showed that NETest could identify residual micro and macroscopic disease in patients who underwent surgery. More specifically, on post-operative day 30 a NET score > 20 was predictive of radiologic disease recurrence with an accuracy of 94% [49]. In another study by Bodei et al., NETest correlated accurately even with treatment modalities beyond surgery such as Peptide receptor radionuclide therapy (PPRT) and was found to be an accurate marker of response to PRRT [50]. The overall utility of NETest for both diagnostic and predictive purposes was depicted in a recent meta-analysis by Oberg et al. In their study Oberg et al. found that NETest was 90.2–93.6% accurate as a type 0 marker (natural disease history), 84.5–85.5% accurate in differentiating stable disease from progressive disease and 93.7–97.4% accurate as type II biomarker, providing evidence of treatment effect on surrogate endpoints [45].

Despite being one of the most promising available NET biomarkers, there are still shortcomings that should be considered. First, available data are still limited and larger multi-center cohorts that will evaluate NETest performance are needed. Future prospective studies are needed to evaluate NETest performance on specific NET types (including SI-NETs) as well as different tumor grades and stages. In addition, NETest availability and cost remain a concern, however, recently published cost effectiveness estimates seem to be encouraging [51].

#### 3.2.2. Circulating Tumor Cells (CTCs)

CTCs have been proposed as potentially promising prognostic NET biomarkers [52]. CTCs can be differentiated by other cells based on their size as well as the expression of special molecules named epithelial adhesion molecules (EpCAMs) and they are detected by specific molecular assays such as the Cell Search (Veridex) [53]. Diagnostically-wise CTCs seem to perform poorly in midgut NETs. For example, in a study by Khan et al. CTCs were detected only in 43% of patients with metastatic midgut NETs [53]. Their role is mostly confined on NET disease progression, and in 2015 a consensus statement for the need for NET biomarkers recommended further studies to confirm the prognostic value of CTC prognosis [28]. Although the data remain limited, a recent study showed that CTCs detected by Cell Search can accurately predict outcomes in midgut NETs. More specifically, Mandair et al. showed that a threshold a CTC threshold of 2 was predictive for progression at 12-month (OR 5.88) death at 36 months (OR 5.09) and progression free survival (HR 2.25) [54]. In addition, when Khan et al. examined the association of posttreatment CTCs with OS in 138 patients with metastatic NENs, 81 of whom had a primary midgut NET, they found that patients with 0 CTCs before and after treatment as well as those with > 50% CTCs reduction had increased OS [55]. Despite showing some promising results, CTCs have several limitations which have not yet allowed their implementation in every day clinical practice, including their inability to identify all tumor cells, the inherent limitations and complexity of analyzing single tumor cells, as well as the uncertain cut off values [11]. As a result, CTCs are not considered a reliable biomarker in terms of diagnosis and management of NENs yet [28].

#### 3.2.3. Circulating Tumor DNA (ctDNA) and Cell Free DNA (cfDNA)

ctDNA (DNA released by tumor cells) and cfDNA (free circulating DNA not necessarily from tumor cells) are DNA fragments that can be detected in blood sample and may play an important role as “liquid biopsies”. Their main role is confined to characterizing the tumor genetic profile with potential implications in personalized, targeted therapies. Although ctDNA and cfDNA have been studied in other cancers, they have not been extensively studied in patients with NENs. Most data available are confined to patients with pancreatic NET and gastrointestinal NECs [56,57,58] while there are no available data specific for SI-NETs. Other than experimental, there is currently no role for ctDNA and cfDNA in the management of patients with SI-NET.

#### 3.2.4. Micro RNA (miRNA)

miRNA comprise a family of none coding RNAs that play an important role in gene regulating carcinogenesis and have been implicated in the pathogenesis of neoplasia [59]. Circulating miRNAs have been proposed as NET biomarkers. In terms of diagnosing SI-NETs, Malczewska et al. found that a 4-miRNA combination (miR-125b-5p, miR-362-5p, miR-425-5p, and miR-500a-5p) was able to differentiate SI-NET patients from healthy controls with an AUC of 0.951 [60]. From a predictive standpoint, authors of the aforementioned study found that miR-125b-5p correlated well with residual disease after surgical resection, while miR-362-5p seemed to be up-regulated in case of residual disease or recurrence. In their study Miller et al. found that miR-204-5p, miR-7-5p and miR-375 were the most commonly upregulated miRNAs in 90 patients with progressive SI-NETs [61], while Bowden et al. found that a combination of increased miR-22-3p and miR-21-5p and low miR-150-5p levels were associated with metastatic SI-NETs and worse OS [62]. As seen above, micro-RNA system remains complicated and the identification of an accurate and disease specific miRNA biomarker will likely require a combination of multiple miRNA signatures [63]. It is also unclear if other parameters (e.g., treatment with somatostatin analogs) affect the levels of miRNA [64]. Despite increasing interest in using micro-RNA as a biomarker in small bowel and other neuroendocrine neoplasia, data remain scarce.

## 4. Biomarkers in the Diagnosis and Management of Carcinoid Heart Disease

**Figure 2:**
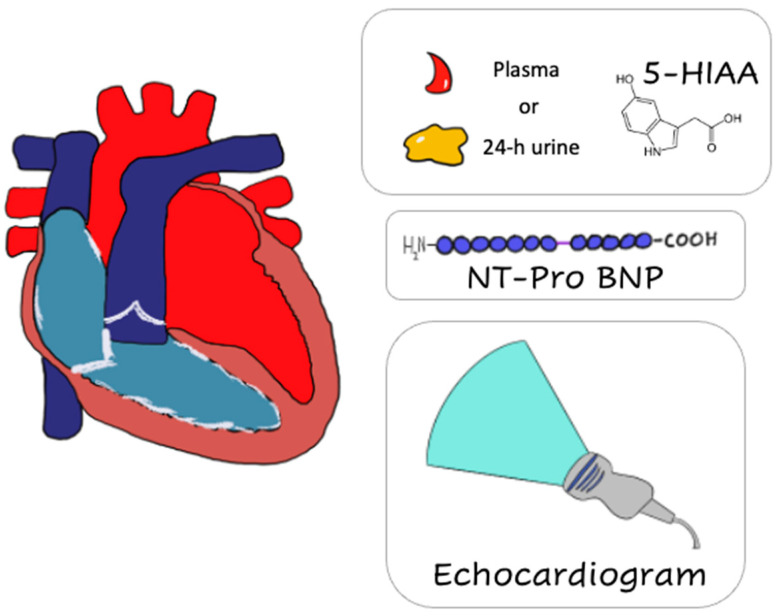
Main diagnostic modalities for the screening and diagnosis of Carcinoid Heart Disease. NT-pro BNP, N-terminal pro–B-type natriuretic peptide; 5-HIAA, 5-hydroxy-indole acetic acid.

Similar to SI-NETs, symptoms of CHD often remain indolent until late in the disease course when signs of right sided heart failure (e.g., peripheral edema) become evident. As a result, increased surveillance, especially in patient with known NET liver metastasis and carcinoid syndrome, is required. Two dimensional (2D) transthoracic echocardiogram (ECHO) remains the gold standard in diagnosis and follow-up of patients with known or suspected CHD. More advanced imaging studies (such as 3D ECHO and cardiac MRI) can always be utilized for better visualization of difficult-to-view heart structures such as pulmonary valves. Similar to SI-NETs, biomarkers play an important role in the diagnosis and management of CHD. In this section of our review, we discuss the biomarkers utilized as diagnostic, prognostic and predictive tools in patients with CHD (Figure 2).

### 4.1. Serotonin and Metabolites

It is thought that serotonin along with other growth factors (such as TGFb) and peptides secreted by NETs are the major etiologies of fibrosis seen in CHD [15]. Serotonin can be measured by different means including blood serotonin, plasma 5-HIAA, or 24-h urine 5-HIAA. Usually, those markers are elevated in carcinoid syndrome but are significantly higher in patients with CHD. Measurement of either plasma or 24-h urine 5HIAA is necessary for the diagnosis and follow up of CHD, while increase levels can also identify patients at high-risk for developing CHD. In their study Bhattacharyya et al. found that a urine 5-HIAA level ≥ 300 μmol/24 h was an independent predictor for development or progression of CHD [65]. Plasma 5-HIAA has also been found to correlate well with urinary 5-HIAA and can potentially serve as an easier alternative biomarker for CHD development and progression [14,29,66,67]. US guidelines suggest that patients with increased levels of 5-HIAA should undergo annual echocardiography [68]. In addition, highly elevated levels of urine 5-HIAA along with echocardiographic evidence of CHD could be an indication for use of Telotristat, a tryptophan hydroxylase inhibitor [68]. In a recent review that included 31 studies, Buchanan-Hughes et al. reported that higher 5-HIAA levels are associated with both disease progression and increased mortality in patients with CHD [69]. 5-HIAA pitfalls are similar to the ones described in the SI-NETs section.

### 4.2. Chromogranin A

Chromogranin is the most commonly used mono-analyte biomarker for NETs. Although CgA is almost always elevated in the setting of CHD, the use of this biomarker in the diagnosis of CHD is not recommended due to poor specificity. In terms of prognosis, previous studies have reported that concomitant elevation of N-terminal pro–B-type natriuretic peptide (NT-pro BNP) and CgA is associated with increased mortality in patients with CHD [70].

### 4.3. Activin A

Activin A belongs to the TGF family and can be used as a predictor of CHD development. Using a cut off of ≥ 0.34 ng/mL, Activin A had 87% sensitivity and 57% specificity for detecting CHD [71]. However, disease state cannot be accurately differentiated based on this biomarker.

### 4.4. Connective Tissue Growth Factor for Carcinoid Heart Disease (CTGF/CCN2)

CTGF/CCN2 is a mono-analyte biomarker that has been associated with right ventricle dysfunction and valve regurgitation in patients with NETs. In a study by Bergestuen et al., patients with higher plasma CCN2 levels were more likely to have reduced right ventricle function with a sensitivity of 88% and specificity of 69% [72]. However, the use of CTGF/CCN2 has not been established in clinical practice.

### 4.5. N-Terminal Pro–B-Type Natriuretic Peptide (NT-Pro BNP)

**Figure 3:**
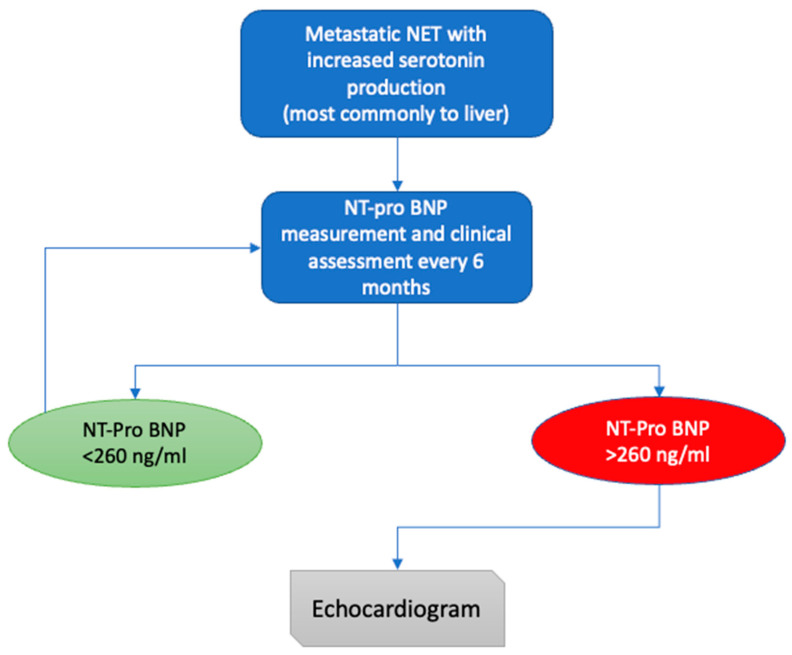
Proposed algorithm for screening and diagnosis of CHD (adopted by Davar et al. [14]) NT-pro BNP, N-terminal pro–B-type natriuretic peptide.

NT-pro BNP is a sensitive and specific marker for detection, disease progression and survival of patients who develop CHD secondary to carcinoid syndrome. In terms of detection, previous studies suggest a cut-off level of 260 pg/mL as a screening tool in patients with carcinoid syndrome. The aforementioned cut-off has a sensitivity of 92% and specificity of 91% for detection of CHD [73]. As a result, NT-pro-BNP is currently considered the best biomarker available for CHD screening in patient with carcinoid syndrome. Patients with metastatic NETs should have NT-pro BNP measured every 6 months, followed by transthoracic echocardiogram for those with NT-pro BNP levels above 260 pg/mL (Figure 3) [14]. High levels of NT-pro BNP have also been found to correlate with worse OS in patients with CHD and Dobson et al. reported that for every 100 ng/mL increase in NT-proBNP, mortality risk was also increased by 11% [74,75].

## 5. Conclusions

In this review we delineated the wide spectrum of biomarkers used in the field of GEP-NETs with emphasis placed on SI-NETs and CHD. We highlighted that there is a growing armamentarium of molecules with potential diagnostic and prognostic utility, especially in the area of multi-analyte assays like NETest, which aim to surpass the inherent pitfalls of mono-analyte biomarkers like CgA and 5-HIAA by offering consistent and reproducible results, in addition to superior sensitivity and specificity. However, their efficacy remains to be validated by additional studies, with the use of traditional biomarkers still predominating. Based on the available data and until NETest are further validated and become widely available, traditional mono-analytes can be used as adjunct to diagnosis and prognosis. More specifically for SI-NETs CgA should be used and interpreted with caution, given that diagnostic accuracy is limited, while 5HIAA should be followed especially for surveillance of carcinoid syndrome. In addition, the use of biomarkers in the field of CHD is more or less extrapolated from experience with GEP-NETs and carcinoid syndrome in general. For CHD, 5-HIAA and NT-proBNP remain the main biomarkers and should be utilized mainly as screening tools for the development and assessment of progression of CHD in patients with carcinoid syndrome. Given that the role of multi-analyte assays is less clear, future studies should assess the role of novel biomarkers in CHD. The above findings indicate that the search for a “one-size-fits-all” gold standard biomarker in the field of GEP-NETs is still ongoing.

## Figures and Tables

**Table 1:** Overview of SI-NETs biomarkers.

Biomarker	Type	Utility in SI-NETs			Comments
		Diagnostic	Predictive	Prognostic	
CgA	Monoanalyte	+	−	+	Lab result variability, impairment by multiple diseases and medications. Diagnostic accuracy: 40–70% [11].
5-HIAA	Monoanalyte	+	−	+	Plasma 5HIAA comparable to 24 h urine 5HIAA. Useful diagnostic and prognostic aid in SI-NETs.
CgB	Monoanalyte	+	−	+	Like CgA but not impaired by renal disease and PPI use.
Pancreastatin	Monoanalyte	+	+	+	Diagnostic accuracy: 40–60% [11]. Prognostic accuracy may be superior to CgA.
Neuro-specific enolase	Monoanalyte	−	−	+	Minimal utility in SI-NET.
Neurokinin A	Monoanalyte	−	−	+	Minimal utility in SI-NET.
NETest	Multianalyte	+	+	+	Great diagnostic, predictive and prognostic value. Cost and availability remain a concern.
CTCs	Multianalyte	−	+	−	Not widely available, still experimental. No diagnostic value while prognostic value is unclear.
MiRNA	Multianalyte	+	+	+	Promising but not widely implemented. Limited available data.

SI-NET, small intestine neuroendocrine tumors; CgA, chromogranin A; CgB, chromogranin B; 5-HIAA, 5-hydroxy-indole acetic acid; CTCs, circulating tumor cells; MiRNA, micro RNA; PPI, proton pump inhibitors.
